# Social wellbeing in older breast cancer survivors taking adjuvant endocrine therapy: impacts of self-efficacy for symptom management and pain

**DOI:** 10.3389/fragi.2026.1831434

**Published:** 2026-05-11

**Authors:** Nicole A. Arrato, Addison D. Monroe, Francis J. Keefe, Gretchen G. Kimmick, Rebecca A. Shelby

**Affiliations:** 1 Duke University School of Medicine, Department of Psychiatry & Behavioral Sciences, Durham, NC, United States; 2 Supportive Care and Survivorship Center, Duke Cancer Institute, Durham, NC, United States; 3 Memorial Sloan Kettering Cancer Center, Department of Psychiatry & Behavioral Sciences, New York, NY, United States; 4 Duke University School of Medicine, Department of Medicine, Durham, NC, United States

**Keywords:** adjuvant endocrine therapy, breast cancer, older adults, pain interference, self-efficacy, social wellbeing

## Abstract

**Introduction:**

For older breast cancer survivors taking adjuvant endocrine therapy (AET), side effects are common and significantly impact quality of life. Self-efficacy is an important protective factor in this population, but little is known about its relationship to pain interference and social wellbeing. This study examined the relative contributions of self-efficacy for managing symptoms (SEMS) to objective and subjective pain interference and social wellbeing.

**Methods:**

Fifty-one women aged ≥65 taking AET for breast cancer completed measures at the time of routine medical follow-up. The measures assessed SEMS, pain intensity, pain interference (subjective report and objective measurement, i.e., the timedup and go test), and social wellbeing.

**Results:**

Hierarchical regression analyses demonstrated that SEMS was significantly associated with social wellbeing, over and above pain-related variables. When examining pain interference as a subjective measure, pain interference was initially associated with social wellbeing (*p* = 0.001), but was no longer significant (*p* = 0.443) when SEMS (*p* < 0.001) was added into the model. When examining pain interference via objective measure, only SEMS was significantly associated (*p* < 0.001) with subjective and objective measures of pain interference and social wellbeing, over and above other factors.

**Discussion:**

Results highlight the importance of SEMS as a pathway to preserve social wellbeing and potentially minimize loneliness in older breast cancer survivors. Interventions which prioritize SEMS and address social wellbeing may also have downstream impacts on AET adherence, recurrence rates, and quality of life.

## Introduction

Older breast cancer survivors receiving adjuvant endocrine therapy (AET) frequently report side effects (e.g., pain) and loneliness—two interrelated issues which have pronounced negative impacts on quality of life (Burstein et al., 2019; Lemij et al., 2023a; Merdawati et al., 2024; Pilleron et al., 2023; Radwan et al., 2025). Pain is highly prevalent in older adulthood and is often accompanied by functional (Ritchie et al., 2023) and social limitations (Ashton-James et al., 2022; Emerson et al., 2018). In turn, loneliness and poor social wellbeing (an individual’s subjective evaluation of their ability to develop, maintain, and enjoy meaningful relationships) in older adulthood are associated with less activity, higher pain, and poorer physical health, indicating a reinforced cycle (Loeffler and Steptoe, 2021; Macchia and Fett, 2025; Powell et al., 2022). In the context of breast cancer, this cycle may be more pronounced due to higher rates of loneliness, cancer-related social constraints (e.g., avoidance and criticism), AET side effects, and comorbidities (Adams et al., 2018; Li et al., 2025). Specifically, women receiving AET, the most effective available treatment for recurrence of hormone-receptor positive breast cancer (providing up to 50% reduction in recurrence; Burstein et al., 2019), often experience chronic musculoskeletal and joint pain, cramps, stiffness, and reduced physical functioning, which have compounding impacts on social wellbeing (Boonstra et al., 2013; Haidinger and Bauerfeind, 2019; van Londen et al., 2014; Wood et al., 2017).

Hormone receptor-positive breast cancer, which disproportionately impacts older women (Lemij et al., 2023b), is routinely and effectively treated with AET. While the benefits of AET are substantial, the side effects are burdensome (Boonstra et al., 2013; Haidinger and Bauerfeind, 2019; van Londen et al., 2014). More than 70% of older women taking AET experience significant arthralgia, characterized by non-inflammatory joint pain, stiffness, and achiness (Burstein, 2007; Crew et al., 2007; Oberguggenberger et al., 2011). This side effect significantly interferes with daily functioning, including family and social relationships and engagement in recreational activities (Kimmick et al., 2020; Laroche et al., 2017), leading to high levels of distress and medication nonadherence (Boonstra et al., 2013; Murphy et al., 2012; Post et al., 2024). AET-related arthralgia and symptom distress have been associated with depressive symptoms, lower AET adherence, and unfavorable views towards AET (Post et al., 2024). Because of symptom burden, as high as 59% of patients do not take AET as prescribed (Murphy et al., 2012) leading to significantly higher all-cause mortality rates (Zheng and Thomas, 2023). For older women already facing functional (e.g., osteoarthritis) and social limitations, AET-associated side effects are greater and more persistent than in their younger counterparts (Li et al., 2025). Adding further complexity, the American Society of Clinical Oncology (ASCO) currently recommends that AET be taken for 5–10 years (Burstein et al., 2019), requiring significant coping efforts over long periods of time.

Self-efficacy, or confidence in one’s ability to successfully engage in or perform a specific behavior or task (Bandura, 1998), has been identified as an essential factor in explaining unintentional and intentional AET adherence (Kimmick et al., 2015; Toivonen et al., 2021), AET satisfaction (Lambert et al., 2018; Post et al., 2024), symptom coping (Shelby et al., 2014), and even depression (Post et al., 2024). In a previous study examining a sample of middle-aged and older women with breast cancer, we found that self-efficacy for managing symptoms (SEMS) was associated with higher functional, emotional, and social wellbeing when controlling for physical symptoms (Shelby et al., 2014). Furthermore, self-efficacy moderated the relationship between physical symptoms and functional and emotional wellbeing. Similarly, in another cross-sectional investigation, SEMS moderated the relationships between symptom distress and depression, AET adherence, and AET satisfaction (Post et al., 2024). Despite the abundance of literature documenting the benefits of self-efficacy in this population, most research emphasizes physical and emotional outcomes. Moreover, studies that have investigated social wellbeing (e.g., Shelby et al., 2014) are limited, have not focused on older adults, have not captured self-efficacy specific to symptom management, and have not examined competing drivers of social wellbeing. For older breast cancer survivors navigating AET-related symptoms, SEMS may enhance valued activity engagement and therefore preserve social behavior and engagement. Conversely, low SEMS may restrict activity and reinforce avoidance, leaving women vulnerable to loneliness.

SEMS–including long-term cancer treatment effects, medication side effects, and menopausal symptoms–is particularly important for older breast cancer survivors receiving AET. Yet the impact of SEMS on social wellbeing is unknown in this population. Pain severity or intensity; objective and subjective pain interference (Emerson et al., 2018; Loeffler and Steptoe, 2021; Powell et al., 2022); medical comorbidities (Trtica et al., 2023); and functional decline (Pollak et al., 2023) are also suspected drivers of social wellbeing in older breast cancer survivors. For example, pain predicted loneliness longitudinally in a sample of aging adults (Emerson et al., 2018). The literature has largely examined these factors in isolation rather than considering how they intersect to predict social outcomes. Identifying modifiable factors that support social wellbeing among older women taking AET is essential for informing psychosocial intervention.

The present study aims to examine associations between SEMS, pain intensity, objective pain interference, subjective pain interference, and social wellbeing in older women receiving AET for breast cancer. We hypothesized that (1) pain intensity and objective and subjective pain interference would be associated with lower social wellbeing; (2) SEMS would be associated with higher social wellbeing; and (3) the associations between pain interference and social wellbeing would still be significant with the inclusion of SEMS. By illuminating the psychosocial factors associated with social wellbeing, this work seeks to inform interventions that will impact both AET symptom burden and loneliness in older women with breast cancer.

## Materials and methods

### Participants

Participants were recruited from the Duke Cancer Institute. This study was approved by the university Institutional Review Board (Pro00016507), and all participants provided informed consent. Individuals were eligible if they met the following inclusion criteria: 1) a diagnosis of Stage I to Stage IIIA breast cancer; 2) hormone receptor positive tumor defined as any positivity of estrogen or progesterone receptor; 3) completed local definitive treatment (i.e., surgical treatment, chemotherapy and/or radiation therapy); 4) post-menopausal, defined as having no ovaries, age >60, or no menstrual period for 1 year in the absence of any hormonal or antihormonal therapy; and 5) taking tamoxifen or an aromatase inhibitor (i.e., anastrozole, letrozole, exemestane) for at least 1 month. Individuals were excluded from the study based on the following criteria: 1) <21 years of age; or 2) unable to provide meaningful consent (i.e., severe cognitive impairment such that descriptions of the research are not clearly understood).

This paper includes *N* = 51 patients over the age of 65 years. Data was part of a larger study (*N* = 114, age range 45–84 years) looking at medication-taking behaviors among women prescribed adjuvant endocrine therapy for breast cancer (Kimmick et al., 2015). See Kimmick et al., 2015, for a description of the larger sample.

### Procedure

Prospective participants were mailed a letter describing the study, and were given a phone number to call to obtain additional information or to decline further contact. Prospective participants were approached by a research assistant in the clinic on the day of an existing appointment. After completing informed consent, participants completed self-report questionnaires and the Timed Up and Go test. Information regarding the participants’ breast cancer history and medical comorbidities was obtained via medical record. Participants were given $10 for participating.

### Measures

#### Demographics

Age, race, education, and marital status were self-reported. For the present study, marital status was coded as a dichotomous variable (currently married vs. not married). Information obtained from medical records included current and past endocrine therapies, information about breast surgery, and information about other treatments received for breast cancer.

#### Comorbidities

The Adult Comorbidity Evaluation (ACE-27; Piccirillo et al., 2004) was used to obtain comorbidities from medical chart review. The ACE-27 is a validated chart-based instrument that grades specific conditions into levels of mild (grade 1), moderate (grade 2), or severe (grade 3) (Kallogjeri et al., 2014; Kimmick et al., 2018). Based on the highest-ranked ailment, individuals were assigned an overall comorbidity score (i.e., none, mild, moderate, or severe). In cases where two or more conditions were rated as moderate in severity, an overall comorbidity score of ‘severe’ was assigned.

#### Pain intensity

Pain intensity was assessed via self-report using the pain intensity/severity subscale of the Brief Pain Inventory-Short Form (BPI-SF; Cleeland, 1989), which is a highly validated measure in breast cancer samples (Andersson et al., 2020; Galiano-Castillo et al., 2016; Ferreira et al., 2015). This subscale assesses pain intensity over the past week and consists of 4 items rated on a 0–10 scale. Items were computed to create an average score, with higher scores indicating greater pain intensity. Cronbach’s alpha was 0.93 in the present sample.

#### Pain interference

Pain interference was assessed via self-report using the pain interference subscale of the Brief Pain Inventory-Short Form (BPI-SF; Cleeland, 1989), which assesses pain interference over the past week. This subcale consists of 7 items rated on a 0–10 scale. Items were computed to create an average score, with higher scores indicating greater pain interference. For the present study, pain interference scores were dichotomized (no pain interference [0] vs. any pain interference [≥1]), in order to provide insight on the impact of any level of pain interference. This is especially relevant for this patient population, as individuals on AET typically report dull, nagging pain, rather than severe pain flares. Even given its clinical relevance, it is important to acknowledge the potential limitations of dichotomizing variables, i.e., reduced variability or statistical power. Cronbach’s alpha was 0.90 in the present sample.

Pain interference was also assessed via an objective measure of physical function, the Timed Up and Go (TUG) test (Podsiadlo and Richardson, 1991). The TUG is a clinical assessment used to measure functional mobility, primarily in older adults. The test is 3 min in length; a patient is timed as they stand from a chair, walk 3 m, turn, return to the chair, and sit down. A total time less than 10 s is interpreted as normal/freely mobile; ≥14 s indicates mobility impairment and high fall risk. The TUG has been used as an indicator of functional interference; prior research has found correlations between TUG scores and pain interference questionnaires (Moreno-Ligero et al., 2024), as well as TUG and pain disability (Buraschi et al., 2022) in pain populations. The TUG has also been correlated with pain and symptom interference specific to the breast cancer population (Sitlinger et al., 2019).

#### Self-efficacy for managing symptoms (SEMS)

To assess patients’ confidence regarding their ability to cope with symptoms, a modified version of a standard self-efficacy scale (Lorig et al., 1989) was used. Patients rated their confidence in their ability to cope with eight symptoms (e.g., aches and pains, hot flashes and sweating) on a 10-point scale ranging from 10 (“not confident”) to 100 (“very confident”). Items were computed to create an average score, with higher scores indicating greater self-efficacy. This assessment has been used to measure self-efficacy in patients with breast cancer in prior research (Kimmick et al., 2015). Cronbach’s alpha was 0.91 in the present sample.

#### Social wellbeing

The Functional Assessment of Cancer Therapy - General (FACT-G; Cella et al., 1993) is a 27-item self-report questionnaire measuring quality of life in cancer patients across four domains: social, physical, functional, and emotional wellbeing. The FACT-G is a very well-validated measure in patients with cancer (Overcash et al., 2001; Sanchez et al., 2011). The present study examined the measure’s social wellbeing domain. The subscale’s 7 items (e.g., “I get support from my friends,” “I am satisfied with family communication about my illness”) are scored on a 5-point scale from 0 (“not at all”) to 4 (“very much”), with higher sum scores indicating greater social wellbeing. Cronbach’s alpha was 0.86 in the present sample.

### Analyses

Descriptive statistics and correlational analyses were conducted. Hierarchical linear regressions were conducted to examine the relationships between SEMS, pain intensity, pain interference (subjective and objective measures), and social wellbeing. Separate regression models were conducted for the subjective measure of pain interference and the objective measure of pain interference, to prevent collinearity. Each regression model included demographic and medical covariates.

## Results

### Sample description

Participant characteristics and descriptive statistics are displayed in Table 1. Participants were on average 71.8 (SD = 5.39) years old; half (51%) were currently married; and approximately half (52%) had a college undergraduate degree or more education. The sample was predominantly White (74.5%). Patients were, on average, 39.3 (SD = 29.95) months post-surgery and 56.9% of women underwent breast-conserving surgery. Approximately one-third of the sample (37.3%) received chemotherapy and 76.5% received radiation therapy. The majority of patients were taking an aromatase inhibitor (78.4%) and the remaining patients (21.6%) were taking tamoxifen. Patients had been taking their current endocrine therapy for an average of 29.7 months (SD = 24.83, range 0–98 months). The majority (47.1%) of the sample had an overall comorbidity score of 1, indicating mild severity of comorbidities.

**TABLE 1:** Sample characteristics.

​	% (*n*)	Mean (SD)	Range
Age	​	71.78 (5.39)	65 to 84
Married	51.0% (26)	​	​
Education			
High school	19.6% (10)	​	​
Some college	27.5% (14)	​	​
College degree	25.5% (13)	​	​
Graduate/Professional school	25.5% (13)	​	​
Race			
White	74.5% (38)	​	​
Black/African American	25.5% (13)	​	​
Cancer stage			
I	39.2% (20)	​	​
II	43.1% (22)	​	​
III	17.6% (9)	​	​
Type of surgery			
Breast conserving surgery	56.9% (29)	​	​
Mastectomy	43.1% (22)	​	​
Months from surgery	​	39.27 (29.95)	2.79 to 125.70
Received chemotherapy	37.3% (19)	​	​
Received radiation therapy	76.5% (39)	​	​
Type of Endocrine Therapy			
Aromatase inhibitor	78.4% (40)	​	​
Tamoxifen	21.6% (11)	​	​
Months on current endocrine therapy	​	29.69 (24.83)	0 to 98
Months on any endocrine therapy	​	35.47 (31.44)	1.15 to 130.30
Adult comorbidity evaluation	​	1.18 (0.93)	0 to 3
0: None	23.5% (12)	​	​
1: Mild	47.1% (24)	​	​
2: Moderate	17.6% (9)	​	​
3: Severe	11.8% (6)	​	​
Pain intensity (BPI-SF)	​	1.66 (2.29)	0 to 8.50
Pain interference (BPI-SF)	​	1.43 (1.84)	0 to 6.86
Timed up and go	​	10.76 (3.23)	6.00 to 21.00
Self-efficacy for managing symptoms	​	75.08 (20.92)	10.00 to 100.00
Social wellbeing (FACT-G)	​	22.70 (6.23)	5.83 to 28.00

### Associations between participant characteristics and study variables

Bivariate analyses were conducted to examine the relationships between participant characteristics and study variables. Younger age was associated with receiving chemotherapy (*r* = −0.417, *p* = 0.002). More severe medical comorbidities were associated with worse performance on the TUG test (*r* = 0.561, *p* < 0.001). Having undergone radiation therapy was associated with lower pain interference (*r* = −0.287, *p* = 0.041) and with greater SEMS (*r* = 0.392, *p* = 0.005). Of note for this patient population, longer length of time on AET was significantly correlated with more severe pain interference (*r* = 0.316, *p* = 0.027). Education level and marital status were not significantly (*p* < 0.05) related to any study variables.

### Correlations among study variables

Table 2 displays correlations among the study variables. Greater subjective pain interference was associated with lower SEMS (*r* = −0.433, *p* = 0.002), and worse social wellbeing (*r* = −0.408, *p* = 0.003). Better performance on the TUG test was associated with greater SEMS (*r* = −0.377, *p* = 0.012) and higher levels of social wellbeing (*r* = −0.336, *p* = 0.022). Additionally, higher SEMS was significantly correlated with greater social wellbeing (*r* = 0.679, *p* < 0.001).

**TABLE 2:** Correlations among study variables.

​	Pain intensity	Pain interference, subjective	Pain interference, objective	Self-efficacy for managing symptoms	Social wellbeing
Pain intensity (BPI-SF)	1.00	​	​	​	​
Pain interference, subjective (BPI-SF)	0.81*	1.00	​	​	​
Pain interference, objective (TUG)	0.34*	0.29*	1.00	​	​
Self-efficacy for managing symptoms (SEMS)	−0.27	−0.43*	−0.38*	1.00	​
Social wellbeing (FACT-G)	−0.29*	−0.41*	−0.34*	0.68*	1.00

*p < 0.05.

### Hierarchical regression analyses

Tables 3 and 4 display the hierarchical linear regression results. The first hierarchical regression examined the relationship between patients’ subjective, self-reported experience of pain interference (BPI Pain Interference subscale) and social wellbeing (FACT Social Wellbeing subscale). Step 1 of the model included marital status and comorbidities (ACE-27), neither of which were significant contributors to social wellbeing. In Step 2, pain intensity and pain interference were added into the model, in order to determine the impact of these constructs in particular. In this step, pain interference was the only significant contributor to social wellbeing (*p* = 0.001). In Step 3, SEMS was added into the model, to explore its specific association with social wellbeing, relative to pain. In doing so, SEMS became the only significant predictor (*p* < 0.001), with pain interference no longer significant (*p* = 0.443).

**TABLE 3:** Hierarchical linear regression examining impacts on social wellbeing, including subjective pain interference per the Brief Pain Inventory (BPI) pain interference subscale.

​	*B*	SE	*t*	*p*
Step 1				
Marital status	1.45	1.74	0.84	0.41
Comorbidities	−1.25	0.95	−1.32	0.19
Step 2				
Marital status	1.50	1.52	0.99	0.33
Comorbidities	−0.53	0.87	−0.61	0.55
Pain interference (BPI)	−6.73	1.97	−3.42	0.001*
Pain intensity	0.16	0.44	0.36	0.72
Step 3				
Marital status	1.46	1.30	1.12	0.27
Comorbidities	−0.25	0.75	−0.33	0.74
Pain interference (BPI)	−1.63	2.10	−0.78	0.44
Pain intensity	−0.17	0.38	−0.45	0.66
Self-efficacy for managing symptoms	0.17	0.04	4.11	<0.001*

**TABLE 4:** Hierarchical linear regression examining impacts on social wellbeing, including objective pain interference per the Timed Up and Go (TUG) test.

​	*B*	SE	*t*	*p*
Step 1				
Marital status	0.36	1.76	0.21	0.84
Comorbidities	−0.94	1.03	−0.91	0.37
Step 2				
Marital status	−0.34	1.71	−0.20	0.84
Comorbidities	0.22	1.20	0.18	0.86
Pain interference (TUG)	−0.48	0.34	−1.42	0.16
Pain intensity	−0.55	0.43	−1.28	0.21
Step 3				
Marital status	0.70	1.35	0.52	0.61
Comorbidities	−0.18	0.94	−0.19	0.85
Pain interference (TUG)	−0.02	0.28	−0.08	0.94
Pain intensity	−0.26	0.34	−0.77	0.45
Self-efficacy for managing symptoms	0.17	0.03	5.10	<0.001*

The second hierarchical regression examined the relationship between an objective indicator of pain interference (TUG test) and social wellbeing. As in the first model, Step 1 included marital status and comorbidities (ACE-27), neither of which was a significant contributor to social wellbeing. In Step 2, pain intensity and the TUG were added into the model, neither of which was a significant contributor (*p* = 0.163 and *p* = 0.209, respectively). In Step 3, as SEMS was added into the model, SEMS accounted for a significant variance in social wellbeing (*p* < 0.001).

## Discussion

The present study examined the relationship between SEMS, objective and subjective pain variables, and social wellbeing in a sample of older breast cancer survivors receiving AET. We found that SEMS was significantly associated with social wellbeing, over and above pain-related variables. When examining pain interference via subjective measure, pain interference was initially associated with social wellbeing, but was no longer significant when SEMS was included. When examining pain interference via objective measure, only SEMS was significantly associated with social wellbeing, over and above other factors. At no point did pain intensity play a significant role in determining social wellbeing for this patient sample. This aligns with findings that women taking AET generally report their pain experience as consistent but mild-to-moderate in severity (Wood et al., 2017), rather than involving severe spikes in severity. Overall, results highlight the importance of SEMS as a pathway to preserve social wellbeing and potentially minimize loneliness in older breast cancer survivors.

Interestingly, marital status and the presence of comorbidities had no significant relationships with social wellbeing. In a large sample of older women with breast cancer, unmarried women had worse diagnosis, treatment, and survival outcomes, presumably as the consequence of reduced social support and wellbeing (Osborne et al., 2005). The presence of comorbidities has also previously been demonstrated as a direct and indirect predictor of social wellness or loneliness in older adults (Trtica et al., 2023). However, AET has been associated with multimorbidity (e.g., cardiovascular disease; Kim et al., 2022), which may complicate this relationship. Based on our results, it is possible that the influence of marital status and medical comorbidities is small in comparison to SEMS, which supports prosocial coping in the face of symptom burden.

Unexpectedly, subjective pain interference was not associated with social wellbeing after accounting for SEMS, and objective pain interference was unrelated to social wellbeing even before the inclusion of SEMS. Pain and pain interference are common in older women, especially breast cancer patients receiving AET (Wood et al., 2017), and have been shown to restrict activity (Post et al., 2024; Ritchie et al., 2023; Shelby et al., 2014) and even predict loneliness longitudinally (Emerson et al., 2018). In this context, however, SEMS may attenuate the impact of perceived pain interference by promoting adaptive coping, enabling more social engagement (Brister et al., 2006; Turner et al., 2005). Moreover, the lack of a relationship between objective pain interference and social wellbeing is consistent with the notion that functional limitations alone are insufficient without considering how one appraises such limitations (Pelletier et al., 2020). Given our findings, the powerful influence of SEMS may promote adaptive coping for pain in such a way that is protective against loneliness, irrespective of pain interference levels.

Self-efficacy may be particularly important in the aging breast cancer survivor population. Research in younger breast cancer survivors suggests that symptom and pain interference have stronger ties with social and emotional outcomes, likely due to competing life roles (e.g., employment, caregiving; Avis et al., 2005; Howard-Anderson et al., 2012; Moye et al., 2014). Older adults, on the other hand, often expect age-related pain (Collis and Waterfield, 2015), and are more likely to rely on internal coping resources like self-efficacy (Mosher et al., 2010). This may help to explain why self-efficacy was strongly associated with social wellbeing in the present study, compared to younger adults whose pain interferes more with engagement in activities (Moye et al., 2014). Our findings indicate that self-efficacy may be especially important for symptom management in aging women - perhaps even more essential than the symptoms themselves, or, the functional consequences of symptoms.

Self-efficacy has been uncovered as an essential modifiable factor for women taking AET. To date, self-efficacy has demonstrated significant associations with AET adherence (Kimmick et al., 2015; Toivonen et al., 2021), AET satisfaction (Lambert et al., 2018; Post et al., 2024), symptom coping (Shelby et al., 2014), and even depression (Post et al., 2024) in older women with breast cancer. To our knowledge, this is the first study examining social wellbeing, not physical or emotional wellbeing, as an outcome of SEMS in older breast cancer survivors. Results highlight the importance of self-efficacy, and social-cognitive theory as an extension, in understanding social wellbeing in the aging breast cancer population. In a review of self-management strategies for AET side effects, Hall et al. (2022) noted the significant lack of available evidence-based interventions. In the development and adaptation of self-management strategies going forward, researchers and clinicians should prioritize the facilitation of SEMS in order to protect against loneliness. Specifically, enhancing SEMS may facilitate engagement in adaptive coping, therefore preserving social activity participation (Karademas et al., 2023). Taken together with previous findings, self-efficacy intervention may support behavioral (e.g., adherence), emotional, and social domains of wellbeing.

### Strengths and limitations

The present study has strengths which warrant discussion. Breast cancer survivors face significant functional and social limitations which are exacerbated in the face of aging and AET side effects like pain. Thus, this study captures the unique, compounding barriers faced by this population which will inform targeted interventions to promote social wellbeing. Moreover, social outcomes are largely neglected in the aging and AET side effect literature, leaving a substantial research gap that this study helps to fill. Specifically, the competing influences of SEMS, pain intensity, and pain interference in explaining social wellbeing have not been examined prior to the present study.

This study also has limitations which provide direction for future research. This investigation included a cross-sectional design; thus, longitudinal and experimental designs are needed to explore causality. The study’s relatively small sample size (*N* = 51) is a limitation in relation to the number of predictors included in the regression models; a larger sample size would reduce the likelihood of model overfitting and would address high variance or standard errors. Furthermore, the present sample included primarily White women recruited from a singular medical center, restricting generalizability substantially. Future investigations should focus on enhancing sample heterogeneity to substantiate findings in diverse populations. Lastly, the present sample primarily included women taking aromatase inhibitors (78.4%) which is more often accompanied by pain in comparison to those taking tamoxifen (21.6%; Garreau et al., 2006). Samples of women taking one form of AET over the other may yield different results.

## Conclusion

For older breast cancer survivors taking AET, side effects are common and significantly impact quality of life. To date, the majority of research on this population has focused on physical and emotional outcomes. Self-efficacy has been found to be a particularly important protective factor in this population, but little is known about its relationship to social wellbeing, especially when compared to other notable contributors like pain interference. In the present cross-sectional investigation, we sought to explore the relative contributions of SEMS, pain intensity, and objective and subjective pain interference in explaining social wellbeing. We found that SEMS was significantly associated with social wellbeing, beyond the contributions of pain-related variables. Taken together, results highlight the importance of SEMS as a potential pathway to preserve social wellbeing in older breast cancer survivors. Interventions which prioritize SEMS and impact loneliness may also have downstream consequences on AET adherence, recurrence rates, and quality of life.

## Data Availability

The raw data supporting the conclusions of this article will be made available by the authors, without undue reservation.
